# Amino Acids and Inherited Amino Acid-Related Disorders

**DOI:** 10.1155/2018/5629454

**Published:** 2018-09-10

**Authors:** Ina Knerr, Laurie Bernstein, Ellen Crushell, Siobhan O'Sullivan, Jörn Oliver Sass

**Affiliations:** ^1^National Centre for Inherited Metabolic Disorders, Temple Street Children's University Hospital, Dublin, Ireland; ^2^University College Dublin, Belfield, Dublin, Ireland; ^3^Children's Hospital Colorado, Aurora, USA; ^4^Royal Belfast Hospital for Sick Children, Belfast, UK; ^5^Bonn-Rhein Sieg University of Applied Sciences, Rheinbach, Germany

Amino acids perform multiple essential physiological roles in humans, and accordingly, their importance to health has been the subject of extensive attention. In this special issue of the *Journal of Nutrition and Metabolism*, we focus on the various inborn errors of amino acid metabolism, their diagnostic challenges, new treatment approaches, and recent advances in patient monitoring as well as clinical outcomes.

Inborn metabolic disorders affecting amino acid metabolism are generally rare but are, by their very nature, complex and challenging conditions. Amino acid-related metabolic disorders comprise a very heterogeneous group of disease entities with highly variable presentations. Clinical severity may range from occasional incidental findings in some cases to overwhelming illness, brain damage, or multiorgan involvement in others. Some, but not all, of these conditions are included in regular Newborn Bloodspot Screening programmes. Analysis of amino acids, e.g., in plasma or serum, along with distinctive biochemical markers which may be identifiable by urinary organic acid analysis, depending on the underlying condition, is crucial in the diagnosis and care of patients with inborn errors of amino acid metabolism called aminoacidopathies. Amino acid-related disorders are generally caused by an inborn genetic defect in the metabolic pathways of a particular amino acid or a group of amino acids. Typical examples include phenylketonuria (PKU), maple syrup urine disease (MSUD), or classical homocystinuria (HCU, cystathionine-*β*-synthase deficiency).

We here present exciting original work in the interdisciplinary field of amino acids and related inborn metabolic disorders. It includes, for instance, studies on PKU which constitutes the most common inborn error of amino acid metabolism in humans involving phenylalanine, or on HCU, a metabolic disorder in the metabolic pathway of sulphur-containing amino acids. From a diagnostic viewpoint, age-specific reference intervals for amino acids should be used for a particular population and analysis method, as presented here. We also demonstrate that metabolomic analyses in plasma and urine can serve informative functions in patients with inborn errors of amino acid metabolism.

Overall, the treatment goal for affected individuals is to normalise the striking metabolic imbalance, e.g., at a cellular level and in physiological fluids, as much as possible by implementing, in particular, dietary treatment and medication or cofactor supplementation as appropriate along with patient monitoring and emergency treatment as required. In recent times, advances in diagnostic technology, including expanded Newborn Bloodspot Screening programmes, as well as major advances in treatments, have led to an exciting increase in the body of knowledge regarding amino acid-related disorders which will help to continuously improve our patient outcomes.

In this special issue of the *Journal of Nutrition and Metabolism*, we aim at providing some new insights into the pathophysiological roles of amino acids, diagnostic approaches, patient management, and new treatments of inborn errors of amino acid metabolism with a view to optimising nutritional status and overall patient outcome. Early detection of these conditions in Newborn Bloodspot Screening programmes and early medical intervention and dietetic treatment are major medical achievements; however, there are still ongoing challenges in treating these lifelong conditions which require not only insightful clinical management but also more clinical research and fruitful collaborative outcome studies.

We hope that topics addressed in this special issue of the *Journal of Nutrition and Metabolism* will lead to a better understanding of these conditions as well as further research studies aimed at advancing our knowledge in the fields of inherited amino acid-related disorders. We propose that advanced clinical research will not only offer new insights into the ongoing pathophysiology of these rare disorders but also open up new therapeutic and diagnostic possibilities which over time may lead to improved quality-of-life outcomes in affected individuals.

